# Naoxin’an capsules protect brain function and structure in patients with vascular cognitive impairment

**DOI:** 10.3389/fphar.2023.1129125

**Published:** 2023-04-05

**Authors:** Hui Lu, Mingxi Dang, Kewei Chen, Huajie Shang, Bolong Wang, Shaokun Zhao, Xin Li, Zhanjun Zhang, Junying Zhang, Yaojing Chen

**Affiliations:** ^1^ State Key Laboratory of Cognitive Neuroscience and Learning, Beijing Normal University, Beijing, China; ^2^ Beijing Aging Brain Rejuvenation Initiative Centre, Beijing Normal University, Beijing, China; ^3^ Banner Alzheimer’s Institute, Phoenix, AZ, United States; ^4^ Institute of Basic Research in Clinical Medicine, China Academy of Traditional Chinese Medicine, Beijing, China

**Keywords:** vascular cognitive impairment, traditional Chinese medicine, Naoxin’an capsule, brain function, left middle frontal gyrus, postcentral gyrus

## Abstract

**Introduction:** Vascular cognitive impairment (VCI) is one of the most common types of dementia. Naoxin'an capsule (NXA), a traditional Chinese medicine compound, has been used to treat VCI for a long time in the clinic. Previous studies proved that the NXA capsules could ameliorate the cerebral mitochondrion deficits of VCI animals. This study aimed to investigate the protectiveness of NXA on human brain structure and function in patients with VCI.

**Methods:** In total, 100 VCI patients were enrolled in this 24-week trial and randomly divided into the NXA capsules group (*n* = 50) and the ginkgo biloba capsules control group (*n* = 50). Before and after the treatment, cognitive behavior tests and multimodal brain magnetic resonance imaging were analyzed to comprehensively evaluate the effectiveness of NXA treatment on VCI patients after 24 weeks.

**Results:** We found that the NXA group significantly improved overall cognitive ability (Alzheimer's Disease Assessment Scale-Cognitive section, *p* = 0.001; Mini-Mental Status Examination, *p* = 0.003), memory (Rey-Osterrieth Complex Figure test, *p* < 0.001) and executive function (Trail Making Test-A, *p* = 0.024) performance after treatment compared with the control group. For brain function, the degree of centrality in the left middle frontal gyrus, right postcentral gyrus, and left supplementary motor area increased in the NXA group and decreased in the ginkgo biloba group after treatment. The fractional amplitude of low-frequency fluctuation (fALFF) of the left precentral and right superior parietal gyrus increased, and the fALFF of the right parahippocampal and left inferior temporal gyrus decreased in the NXA group after treatment. For brain structure, the gray matter density of the left postcentral gyrus increased in the NXA group after treatment, and the total volume of white matter hyperintensity showed a decreasing trend but was not statistically significant. Furthermore, the improvement effect of NXA on executive function was associated with changes in brain function.

**Conclusion:** These findings suggest that the NXA capsules improved cognitive performance and multiregional brain function, as well as gray matter structure in the postcentral gyrus.

## 1 Introduction

Vascular cognitive impairment (VCI), the second most common type of dementia, includes mild cognitive impairment to vascular dementia in a broad spectrum of cognitive disorders. Vascular risk factors and cerebrovascular diseases are the predominant cause of VCI (van der Flier et al., 2018; Zhang et al., 2019). Hospitalization and mortality rates were significantly higher in patients with VCI than in those without cognitive impairment (Rockwood et al., 2000). The pathogenesis of VCI was hypothesized as alterations in the blood-brain barrier, altered vascular reactivity, hypoperfusion, and inflammation 4,5.

Currently, the primary intervention for VCI is to treat vascular disease and other associated risk factors to prevent the clinical appearance of VCI (van der Flier et al., 2018), with some controversies (Collins et al., 2002; Shepherd et al., 2002; Biessels and Despa, 2018; Zonneveld et al., 2018). However, not only donepezil and other drugs can improve VCI symptoms (Farooq et al., 2017), but also Traditional Chinese medicine (TCM). TCM has been widely used in clinical practice and has achieved some degree of efficacy in both the prevention and treatment of VCI (Saleem et al., 2008; Koh, 2010; Li et al., 2013; Zhu et al., 2018). Therefore, TCM presents great potential in finding effective therapeutic drugs, and it is critical to explore the capability of TCM to treat vascular cognitive impairment.

Naoxin’an (NXA) capsule as a TCM compound has been used in clinical practice for many years in China. The result shows that NXA benefits and promotes blood circulation, opening vessels, and dredging collaterals. By activating the CREB/PGC-1α pathway, NXA capsules could protect the structure and function of mitochondria, promote antioxidative capacity, and inhibit the resultant oxidative damage (Yu-juan, 2022). NXA capsules can also improve central nervous system inflammatory injury, reduce the release of inflammatory factors, and inhibit hippocampal neuronal damage and apoptosis, thereby improving the learning and memory ability of rats with chronic cerebral ischemia (Guang-Hui, 2021). However, to provide more evidence to prove the utility of NXA capsules in VCI patients, additional clinical trials should incorporate multimodal neural imaging methods.

Ginkgo biloba extract has a long history of being used in Europe and China to alleviate a variety of symptoms related to cognitive impairment (including VCI) with good efficacy (le Bars et al., 1997). Ginkgo biloba contains antioxidative activity, which was proven to restore impaired mitochondrial function (Strømgaard and Nakanishi, 2004). Studies have shown that ginkgo biloba can improve the energy supply, compromised hippocampal neurogenesis, and neuroplasticity (Tchantchou et al., 2007). In addition, ginkgo biloba can also decrease blood viscosity, enhance microperfusion, and increase the level of dopamine in the prefrontal cortex (Yoshitake et al., 2010). In 2019, the Asian Clinical Expert Group on Neurocognitive Disorders recommended ginkgo biloba to use as part of the multidomain intervention for mild cognitive impairment, including underlying cerebrovascular disease by consensus (Kandiah et al., 2019; Kandiah et al., 2021).

According to TCM theory, both NXA capsules and Ginkgo biloba capsules can promote blood circulation, remove blood stasis, and clear collaterals. More importantly, in addition to these, the NXA capsule also can replenish qi, resolve phlegm, and induce resuscitation, which has unique advantages that a single botanical drug does not have. Therefore, the Ginkgo biloba capsule was compared with the NXA capsule in this study.

Based on the pathological mechanisms of VCI, a 24-week randomized controlled clinical trial was designed for VCI patients with ginkgo biloba capsules as the control group. Cognitive behavior tests and multimodal brain magnetic resonance imaging (MRI) technology were used to comprehensively evaluate the effectiveness of NXA capsules in the treatment of VCI in cognitive function, local function properties, gray matter structure, and white matter hyperintensity. Functional and structural adaption following pharmacologic treatment can be demonstrated by non-invasive neuroimaging techniques.

## 2 Methods

### 2.1 Study material

Pharmaceutical ingredients of Naoxin’an capsule: NXA capsule had been approved by the State Food and Drug Administration (institutional approval number: Z20123066) based on trial results in 2017, in China. The composition of Naoxin’an capsules includes Astragalus mongholicus Bunge (Fabaceae; astragali radix, 13.2%), Codonopsis pilosula (Franch.) Nannf (Campanulaceae; Codonopsis radix, 10.24%), Crocus Sativus Linnaeus (Iridaceae; Croci stigma, 8%), Panax notoginseng (Burkill) F.H.Chen (Araliaceae; Notoginseng radix et rhizoma, 8%), Salvia miltiorrhiza Bunge(Lamiaceae; salviae miltiorrhizae radix et rhizoma, 5.3%), Curcuma aromatica Salisbury (Zingiberaceae; Curcumae radix, 5.3%), Pueraria montana var. thomsonii (Benth.) M.R.Almeida (Fabaceae; Puerariae thomsonii radix, 8%), Conioselinum anthriscoides ‘Chuanxiong’ (Apiaceae; chuanxiong rhizoma, 8%), Reynoutria multiflora (Thunb.) Moldenke (Polygonaceae; Polygoni multiflori radix praeparata, 5.3%), Haliotis discus hannai Ino (Haliotidae; Haliotidis concha, 5.3%), Spatholobus suberectus Dunn (Fabaceae; Spatholobi caulis, 5.3%), Gastrodia elata Blume (Orchidaceae; gastrodiae rhizoma, 5.3%), Cinnamomum camphora (L.) J. Presl (Lauraceae; borneolum, 1.3%), Prunus persica (L.) Batsch (Rosaceae; Persicae semen, 5.3%), Arisaema erubescens (Wall.) Schott (Araceae; Arisaema cum bile, 3%), Buthus martensii Karsch (buthidae; Scorpio, 1.3%), Scolopendra subspinipes mutilans L. Koch (scolopendridae; Scolopendra, 1.3%), Moschus berezovskii Flerov (cervidae; mature male Moschus berezovskii Flerov dry secretions, 0.03%) and *Bos taurus* domesticus Gmelin (bovidae; *B. taurus* domesticus Gmelin dry gallstones, 0.53%). The whole production process of the Naoxin’an capsule, from the verification of raw materials to the final product, totally complied with the provisions of Chinese Pharmacopoeia (2020 Edition). The capsules used were produced by China Jilin Yida Pharmaceutical Co. (batch number: 20200608).

Production process of Naoxin’an capsule: Astragalus mongholicus Bunge (Fabaceae; astragali radix) (10.6% of the total), Crocus Sativus Linnaeus (Iridaceae; Croci stigma), Panax notoginseng (Burkill) F.H.Chen (Araliaceae; Notoginseng radix et rhizoma), Pueraria montana var. thomsonii (Benth.) M.R.Almeida (Fabaceae; Puerariae thomsonii radix), Conioselinum anthriscoides “Chuanxiong” (Apiaceae; chuanxiong rhizoma), Gastrodia elata Blume (Orchidaceae; gastrodiae rhizoma), Cinnamomum camphora (L.) J. Presl (Lauraceae; borneolum), Buthus martensii Karsch (buthidae; Scorpio), Scolopendra subspinipes mutilans L. Koch (scolopendridae; Scolopendra), Moschus berezovskii Flerov (cervidae; mature male Moschus berezovskii Flerov dry secretions) and *B. taurus* domesticus Gmelin (bovidae; *B. taurus* domesticus Gmelin dry gallstones) were crushed into fine powder; The Astragalus mongholicus Bunge (Fabaceae; astragali radix) (89.4% of the total), Codonopsis pilosula (Franch.) Nannf. (Campanulaceae; Codonopsis radix), Salvia miltiorrhiza Bunge (Lamiaceae; salviae miltiorrhizae radix et rhizoma), Curcuma aromatica Salisbury (Zingiberaceae; Curcumae radix), Prunus persica (L.) Batsch (Rosaceae; Persicae semen), Reynoutria multiflora (Thunb.) Moldenke (Polygonaceae; Polygoni multiflori radix praeparata), Haliotis discus hannai Ino (Haliotidae; Haliotidis concha), Spatholobus suberectus Dunn (Fabaceae; Spatholobi caulis), and Arisaema erubescens (Wall.) Schott (Araceae; Arisaema cum bile) were boiled with water three times, the first time for 2 h, the second and the third time for 1.5 h respectively; The decoction was combined, filtered, and the filtrate was concentrated to a thick paste with a relative density of 1.30 (50°C). Add the above fine powder, mix, dry, crush into a fine powder, sift, add a proper amount of starch, mix, and then put into capsules.

Pharmaceutical ingredients of Ginkgo biloba capsules: Ginkgo biloba capsules (batch No. 200313) were obtained from Guilin Honghui Pharmaceutical Co., Ltd. (Guilin, China). The composition of Ginkgo biloba capsules is Ginkgo biloba L. (Ginkgoaceae; ginkgo folium).

### 2.2 Study design and participants

This clinical trial has been registered in the Chinese Clinical Trial Registry (ChiCTR-IPR-2100046757) and approved by the Ethics Committee of Basic Research in Clinical Medicine, China Academy of Chinese Medical Sciences (batch No. P21002/PJ02). It was a randomized controlled trial containing an NXA capsule group and a Ginkgo biloba capsule control group, and patients, site investigators, and caregivers were blinded to the treatments. All patients were from Beijing TianTan Hospital and were diagnosed with VCI by at least two neurologists, and all rechecks were conducted by another neurologist. All participants underwent MRI scans and neuropsychological assessment by a professional imaging staff at baseline and 24 weeks after treatment.

The trial inclusion criteria were as follows: 1) patients were between the ages of 45 and 80; 2) they met the diagnostic criteria of VCI as defined by the Diagnostic and Statistical Manual of Mental Disorders, Fifth Edition (DSM-V) (Regier et al., 2009) or National Institute of Neurological Disorders and Stroke-Association Internationale pour la Recherche et l’Enseignement en Neurosciences (NINDS-AIREN) criteria (Román et al., 1993) (see the Supplementary Materials for details); 3) they were able to do normal activities of daily living; 4) they had single or multiple infarct lesions with ischemic stroke or lacunar infarction, and imaging examination showed that the diameter of the lesion was 2–15 mm by; 5) hemorrhages, cortical and watershed infarcts, hydrocephalus, and specific causes of WMLs (e.g., multiple sclerosis) were absent; and 6) they volunteered to participate in the study and signed informed consent forms by the patient themselves or their legal guardians.

The trial exclusion criteria were as follows: 1) physical disabilities, severe aphasia, or any other factor that might prevent completion of the neuropsychological testing; 2) non-dementia-free diseases other than subcortical VCI that might affect cognition; 3) diseases such as inherited or inflammatory small vessel disease, schizophrenia, serious bone, kidney, joint, liver, hematopoietic system, and endocrine system disease as well as cancer; 4) drug or alcohol abuse disorder, or other medications that may affect cognitive function, including anxiolytics, tranquilizers, nootropics, hypnotics, and cholinomimetic agents; and 5) inability to undergo a brain MRI.

One hundred eligible patients were recruited and randomly assigned into the same appearance NXA group (oral NXA capsule) and ginkgo biloba control group (oral ginkgo biloba capsule), which were identical in appearance, 3 times a day for 24 weeks in a 1:1 ratio. Twenty patients dropped out 12 of which were lost to follow-up, 3 had poor compliance, 5 withdrew, but no patients had adverse reactions. Finally, 80 patients (45 in the NXA group and 35 in the ginkgo group) enrolled in the current study (demographic details in Table 1). The inclusion process of participants was shown in Supplementary Figure S1.

**TABLE 1:** Baseline characteristics of participants.

	NXA group (*n* = 45)	Ginkgo group (*n* = 35)	t/*χ* ^2^	*p*
Age	65.76 ± 5.144	65.23 ± 7.765	0.347	0.730
Gender (M/F)	25/20	18/17	0.135	0.713
Education	10.64 ± 3.891	10.14 ± 4.103	0.559	0.578
ADAS-Cog	9.418 ± 3.5543	8.737 ± 2.8755	0.922	0.359
MMSE	27.22 ± 2.653	26.43 ± 1.754	1.528	0.131

Abbreviation: ADAS-cog, alzheimer’s disease assessment scale-cognitive section, MMSE, mini-mental status examination, M = male, F = female.

Of these, 32 participants (16 in the NXA group and 16 in the ginkgo group) completed baseline and follow-up T1-weighted structural MRI imaging scans. Thirty-one participants (15 in the NXA group and 16 in the ginkgo group) completed baseline and follow-up T2-weighed fluid-attenuated inversion recovery (T2w-FLAIR) and resting-state functional MRI (rs-fMRI) imaging scans.

### 2.3 Neuropsychological testing

The primary outcomes were the Mini-Mental State Examination (MMSE) (Folstein et al., 1975) and Alzheimer’s Disease Assessment Scale-Cognitive subscale (ADAS-Cog) (Wang et al., 2004), which covered four domains (general, mental cognitive state, activities of daily living, and behavior) and is considered the gold standard for evaluating the efficacy of anti-dementia treatments (Kueper et al., 2018). Secondary outcomes included performance in four cognitive domains: 1) episodic memory: Rey-Osterrieth Complex Figure (ROCF)-Delay Recall test (Tupler et al., 1995); 2) visual-spatial ability: ROCF-Copy (Tupler et al., 1995); 3) executive function: Trail Making test-A (Gordon, 1972); and 4) language tests: Category Verbal Fluency test (CVFT) (Mok et al., 2004).

### 2.4 MRI data acquisition

Imaging data including T1-weighted structural and resting-state functional scans and T2w-FLAIR images were collected using the Siemens Trio 3T MRI system. High-resolution T1-weighted, sagittal 3D fast-field echo sequences were first obtained, covering the whole brain with the following parameters: 176 slices, echo time = 3.44 ms, repetition time = 1900 ms, slice thickness = 1 mm, inversion time = 900 ms, flip angle = 9°, acquisition matrix = 256 × 256, and the field of view = 256 × 256 mm^2^. Then, gradient echo EPI sequence was used to obtain rs-fMRI scanning, with TR = 2000 ms, flip angle = 90°, TE = 30 ms, slice thickness = 3.5 mm, 36 axial slices, FOV = 200*200 mm, and acquisition matrix = 64 × 64. Finally, the T2w-FLAIR sequence was collected (repetition time = 9000 ms, slice thickness = 3 mm, echo time = 81 ms, flip angle = 150°, and the number of slices = 25) to measure white matter hyperintensities (WMHs).

### 2.5 Structural image preprocessing

T1-weighted images were segmented and spatially registered to the tissue probability maps into the Montreal Neurological Institute (MNI) space using the segmentation routine implemented in the CAT12 (http://dbm.neuro.uni-jena.de/cat12/). Subsequently, we smoothed the gray matter (GM) maps with an 8 mm kernel of full-width-half-maximum. Finally, we estimated the total intracranial volume (TIV) and assessed the quality of the processed images through visual inspection and weighted average image quality index using the quality assurance (QA) framework in CAT12, including only participants whose QA was better than C.

### 2.6 Rs-fMRI data processing

Calculate the amplitude of low-frequency fluctuation (ALFF), fractional ALFF (fALFF), regional homogeneity (ReHo), and degree centrality (DC) using DPARSF (V5.1, http://rfmri.org/DPARSF) (Chao-Gan et al., 2010). The first 10 volumes of each participant were discarded to account for magnetization equilibrium. The EPI images were slice timing corrected and realigned. The head motion threshold was set to the translation by 3 mm and rotation by 3°. Subsequently, the functional images were spatially normalized to the MNI space and resampled to an isotropic voxel size of 3 mm. Finally, we regressed out some nuisance signals from Friston-24 head motion parameters, white matter, and cerebrospinal fluid.

ALFF/fALFF was calculated using filtered signals within the low-frequency range (0.01–0.08 Hz) without additional filtering to examine spontaneous regional brain activity (Xi et al., 2012). Specifically, fALFF was calculated by the ratio of the filtered frequency band (0.01–0.08 Hz) to the entire available frequency band (subject to imaging acquisition). ReHo was computed by Kendall’s consistency coefficient as a measure of the local coherence for the blood oxygen level-dependent (BOLD) signal (Zang et al., 2004), with 27 neighboring voxels without smoothing.

DC quantifies the number of direct connectivity between a given voxel and all other voxels in the voxel-based graphs. Simply put, the time course of each voxel correlates with the time course of all other voxels in the brain. Then, threshold processing was performed for each correlation at r > 0.25 to construct the binary whole-brain functional network of each participant. The threshold was the default setting while calculating the DC map (Buckner et al., 2009). For a given voxel, DC was calculated as the sum of the significant functional connections at the individual level without smoothing.

To simplify cross-patient controls, ALFF, fALFF, ReHo, and DC are converted to Z-scores by subtracting the mean and dividing by the standard deviation within the GM mask. Finally, 29 participants (14 in the NXA group and 15 in the ginkgo group) were enrolled for brain function analysis.

### 2.7 WMH lesion segmentation and quantification

Baseline and follow-up WMHs lesions were automatically segmented using the lesion prediction algorithm as implemented in the LST toolbox (v 2.0.15) for SPM (Schmidt et al., 2012). The toolbox first segments the T1-weighted images into the GM, white matter, and cerebrospinal fluid. This information is then combined with the coregistered FLAIR intensities to calculate lesion belief maps. The 0.5 lesion probability threshold was used to obtain the lesion volume and lesion probability map. No participants were excluded due to image quality problems.

### 2.8 Statistical analysis

The group differences in demographic information and cognitive performance at baseline were examined by two-sample t-tests and χ2 test.

1) To explore the effects of NXA capsules on cognitive function and WMH in patients with VCI, we performed the repeated-measures analysis of covariance (ANCOVA) to assess the interaction effects (controlling for age, education, and sex) on neuropsychological assessment and total WMH volume before and after treatment. A two-sided *p*-value <0.05 was considered statistically significant.
2) To investigate the effects of NXA capsules on brain structure and function in patients with VCI, voxel-based repeated-measures ANCOVA was used to examine whether the NXA group had interactions with the ginkgo group on longitudinal changes in GM density and functional indices, including ALFF, fALFF, ReHo, and DC, after controlling for covariates of age, education, sex, and TIV. To further determine pre- and post-treatment differences, voxel-based paired t-tests were performed in the NXA and ginkgo groups. The significance level was defined as *p* < 0.001 at the voxel level combined with *p* < 0.05 at the cluster level (GRF-corrected).
3) The change in the clustering mean value of the interaction significance index before and after treatment was calculated and partially correlated with cognitive changes (adjusted for age, sex, and education) to examine the association of these neuroimaging measures with clinical outcomes. The significance level was defined as a *p*-value <0.05. SPSS version 22.0 and DPABI were used for statistical analysis.

## 3 Results

### 3.1 Demographics and neuropsychological tests

**FIGURE 1:**
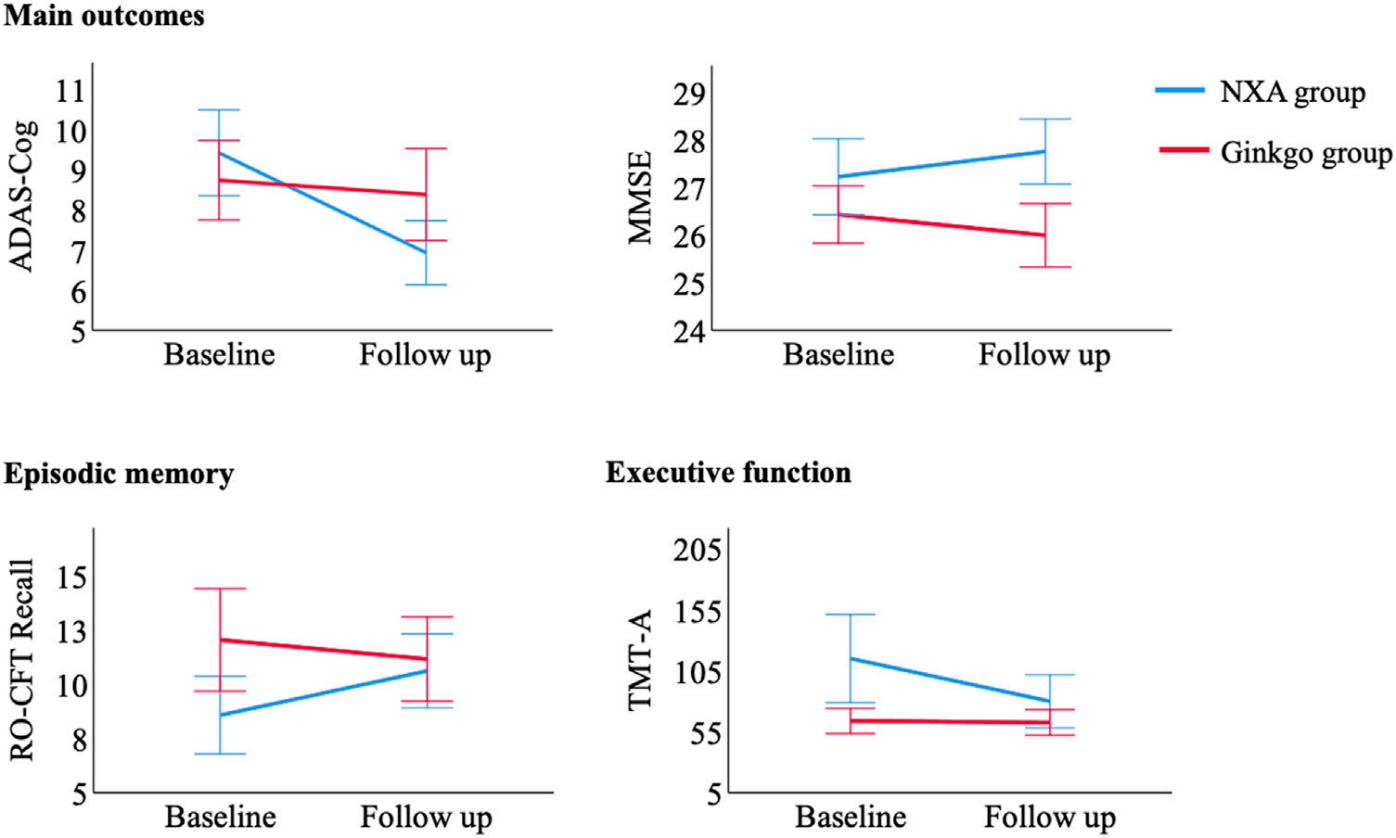
Control of cognitive tests with significant pre- and post-treatment interaction between the NXA group and ginkgo group. Abbreviation: ADAS-cog = Alzheimer’s Disease Assessment Scale-Cognitive subscale; MMSE = Mini-Mental Status Examination; RO-CFT = Rey-Osterrieth Complex Figure test; TMT = Trail Making Test.

There were no significant differences in demographic information and ADAS-cog and MMSE scores between the NXA group and the ginkgo group (Table 1, all *p* > 0.05). For the repeated ANCOVA of cognitive function, significant group × time interaction effects were found in the ADAS-cog (Table 2; Figure 1, *p* = 0.001), MMSE (*p* = 0.003), RO-CFT recall (*p* < 0.001), and TMT-A time (*p* = 0.024). To investigate whether the cognitive improvement effect of the NXA capsule is affected by age and sex, we conducted a supplementary analysis based on median age and sex stratification, respectively. Similar to the results of the overall population, significant group × time interaction was found in the overall cognitive ability and episodic memory function in both younger and older ages (Supplementary Tables S1-S2, *p* < 0.05), and in both males and females (Supplementary Tables S3-S4, *p* < 0.05), without age and sex specificity.

**TABLE 2:** Effect of NXA capsule on cognitive performance in patients with vascular cognitive impairment.

	NXA group		Ginkgo group		Interactions	
	Baseline	Follow up	Baseline	Follow up	F	*p*
Main outcomes						
ADAS-Cog	9.42 ± 3.554	6.93 ± 2.659	8.74 ± 2.876	8.38 ± 3.341	12.606	**0.001**
MMSE	27.22 ± 2.653	27.75 ± 2.267	26.43 ± 1.754	25.99 ± 1.945	9.265	**0.003**
Episodic memory						
AVLT N1-N5	24.84 ± 8.584	27.84 ± 8.504	27.26 ± 7.740	26.94 ± 6.517	0.224	0.638
RO-CFT Recall	8.58 ± 5.971	10.62 ± 5.690	12.06 ± 6.894	11.17 ± 5.675	17.492	**<0.001**
Visual-spatial						
RO-CFT Copy	25.40 ± 6.607	25.01 ± 7.825	30.54 ± 5.548	30.31 ± 5.243	0.156	0.694
Executive function						
TMT-A	99.98 ± 97.101	79.80 ± 69.743	63.77 ± 29.852	62.48 ± 30.404	5.351	**0.024**
Language function						
CVFT	39.47 ± 9.896	38.42 ± 11.470	42.43 ± 8.490	42.41 ± 8.592	0.667	0.417

Statistically significant effects (*p* < 0.05) are in bold.

Abbreviation: ADAS-cog, alzheimer’s disease assessment scale-cognitive subscale; MMSE, mini-mental status examination; AVLT, auditory verbal learning test; RO-CFT, rey-osterrieth complex figure test; CVFT, category verbal fluency test; TMT, trail making test.

### 3.2 Effects of the NXA capsule on brain function

**FIGURE 2:**
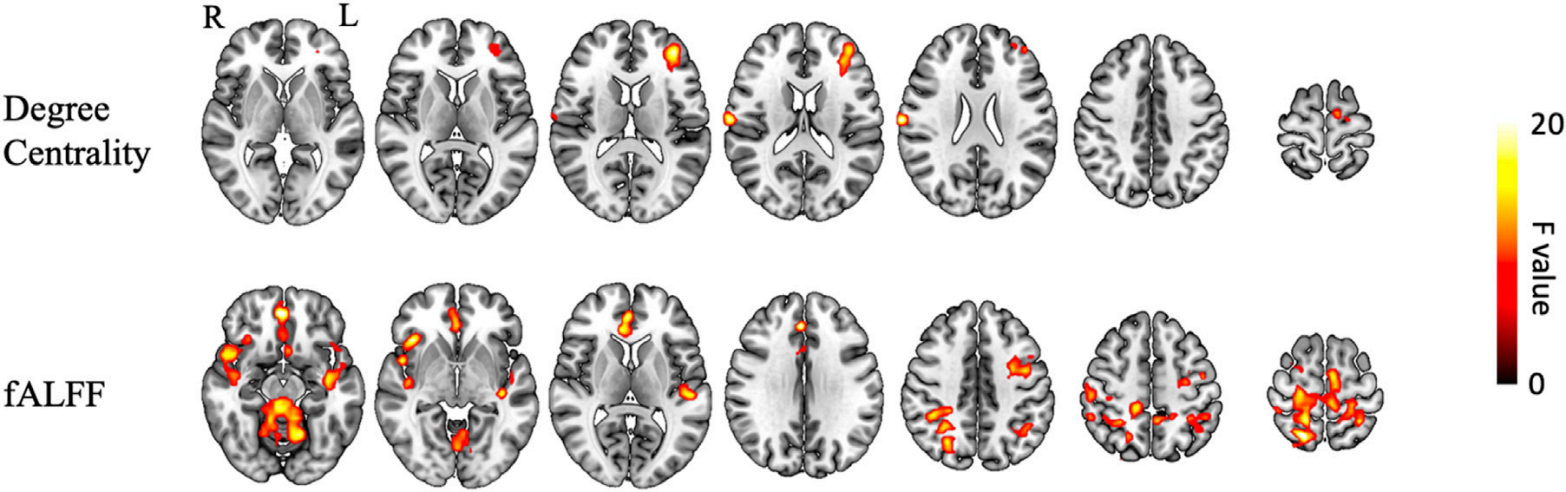
Interaction of brain function between NXA group and ginkgo group before and after treatment. The significance level was set at a voxel-level inference of *p* < 0.001 combined with a cluster-level inference of *p* < 0.05 (GRF-corrected). Abbreviation: fALFF = fractional amplitude of low-frequency fluctuations, L = left, R = right.

**FIGURE 3:**
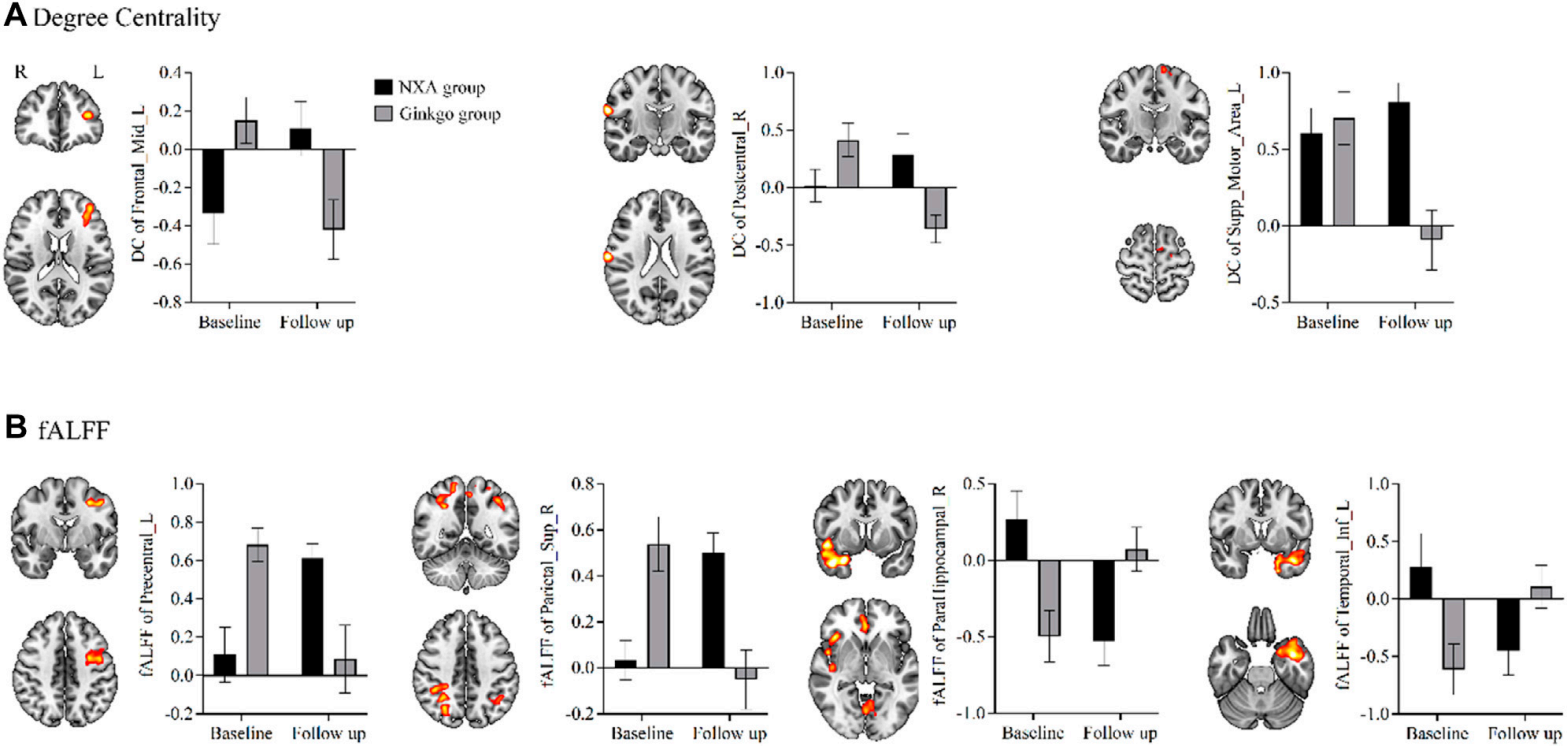
Control of brain function index in regions with significant pre- and post-treatment interaction between NXA group and ginkgo group. **(A)** Effects of the NXA capsule and ginkgo capsule on degree centrality. **(B)** Effects of the NXA capsule and ginkgo capsule on fALFF. Abbreviation: fALFF = fractional amplitude of low-frequency fluctuations, L = left, R = right.

To investigate the effects of the NXA capsules on brain function, voxel-based repeated ANCOVA was used to analyze the changes in DC, fALFF, ALFF, and ReHo indices. We found that the DC of the left middle frontal gyrus, right postcentral gyrus, and left supplementary motor area had a significant group * time interaction effect (GRF-corrected, Figure 2, Supplementary Table S5). Specifically, DC in the right postcentral gyrus, left middle frontal gyrus, and left supplementary motor area increased in the NXA group and decreased in the ginkgo group after treatment (Figure 3A), suggesting that NXA capsules enhance the ability of these regions to transmit information to other brain regions.

For spontaneous regional brain activity, the fALFF of multiple regions had a significant group * time interaction effect (Figure 2, Supplementary Table S5). The fALFF of the left precentral and right superior parietal gyrus increased after treatment in the NXA group and decreased in the ginkgo group (Figure 3B). The fALFF in the right parahippocampal and left inferior temporal gyrus decreased in the NXA group and increased in the ginkgo group after treatment. No significant interaction effect was found between the NXA group and the ginkgo group in ReHo and ALFF before and after treatment.

**FIGURE 4:**
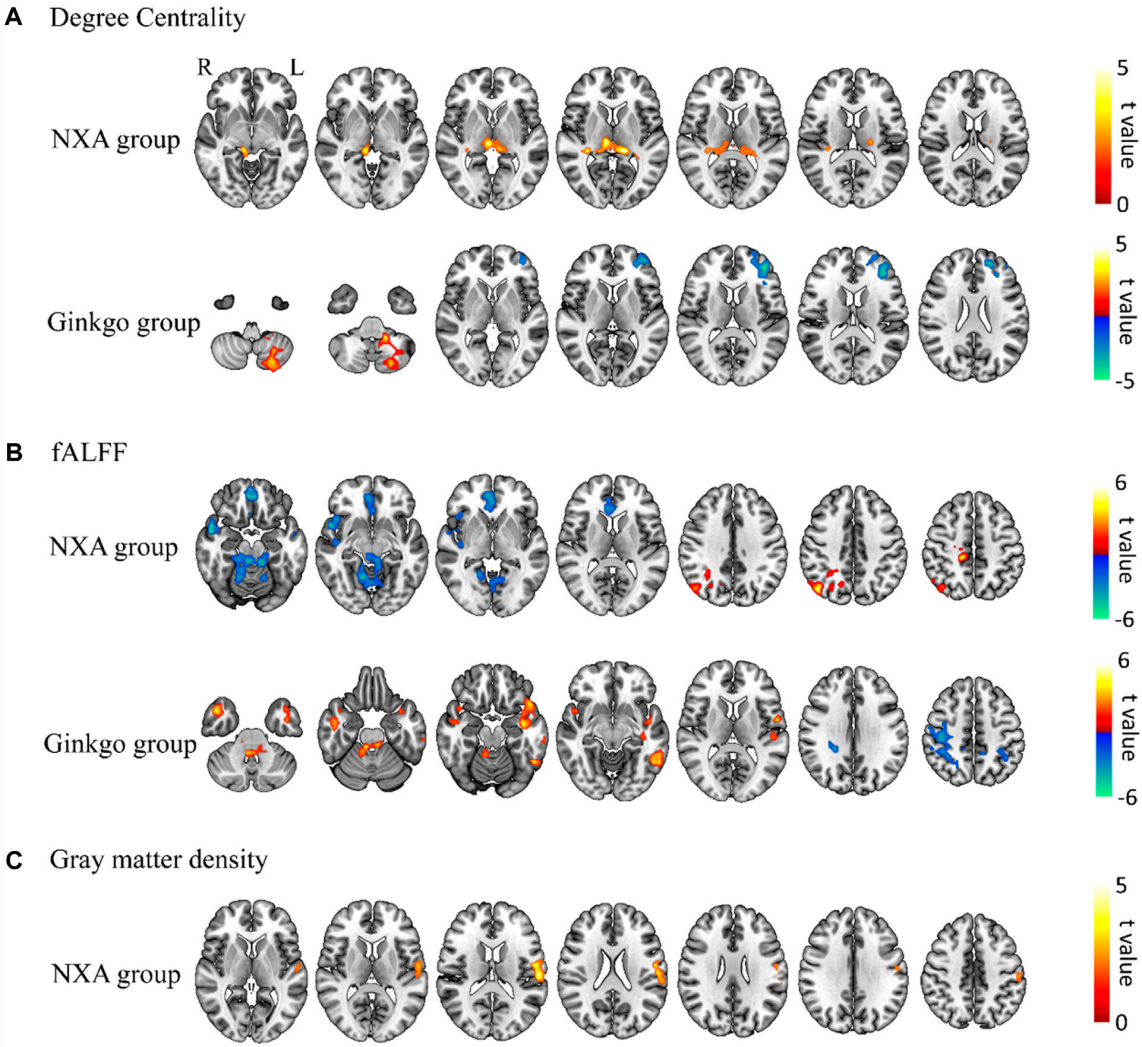
Paired t-test results for brain function and structure for pre- and post-treatment. Changes in degree centrality **(A)**, fALFF **(B)**, and gray matter density **(C)** post-treatment compared with pre-treatment. The significance level was set at a voxel-level inference of *p* < 0.001 combined with a cluster-level inference of *p* < 0.05 (GRF-corrected).

In addition, voxel-based paired t-tests were used to further examine brain function changes after treatment. The DC in the left thalamus increased after treatment in the NXA group, the DC in the inferior frontal gyrus decreased, and the DC in the left cerebellum increased in the ginkgo group (Figure 4A, Supplementary Table S6). The fALFF of the right angular and median cingulate gyri increased, and the fALFF of the right cerebellum, superior temporal gyrus, and orbital part of the middle frontal gyrus decreased in the NXA group after treatment. The fALFF of the cerebellum, right middle temporal gyrus, left rolandic operculum and left inferior temporal gyrus increased, and the fALFF of the right inferior parietal gyrus decreased in the ginkgo group after treatment (Figure 4B, Supplementary Table S7). Similarly, no significant changes were found with ReHo and ALFF.

### 3.3 Effects of NXA capsules on gray matter density

Voxel-based repeated ANCOVA showed that there was no significant interaction between the GM density of the NXA group and the ginkgo group before and after treatment. The results of the paired *t*-test showed that the GM density of the left postcentral gyrus increased in the NXA group after treatment (Figure 4C, Supplementary Table S8), while the GM density of the ginkgo group did not change significantly.

### 3.4 Effects of NXA capsules on white matter hyperintensity

**FIGURE 5:**
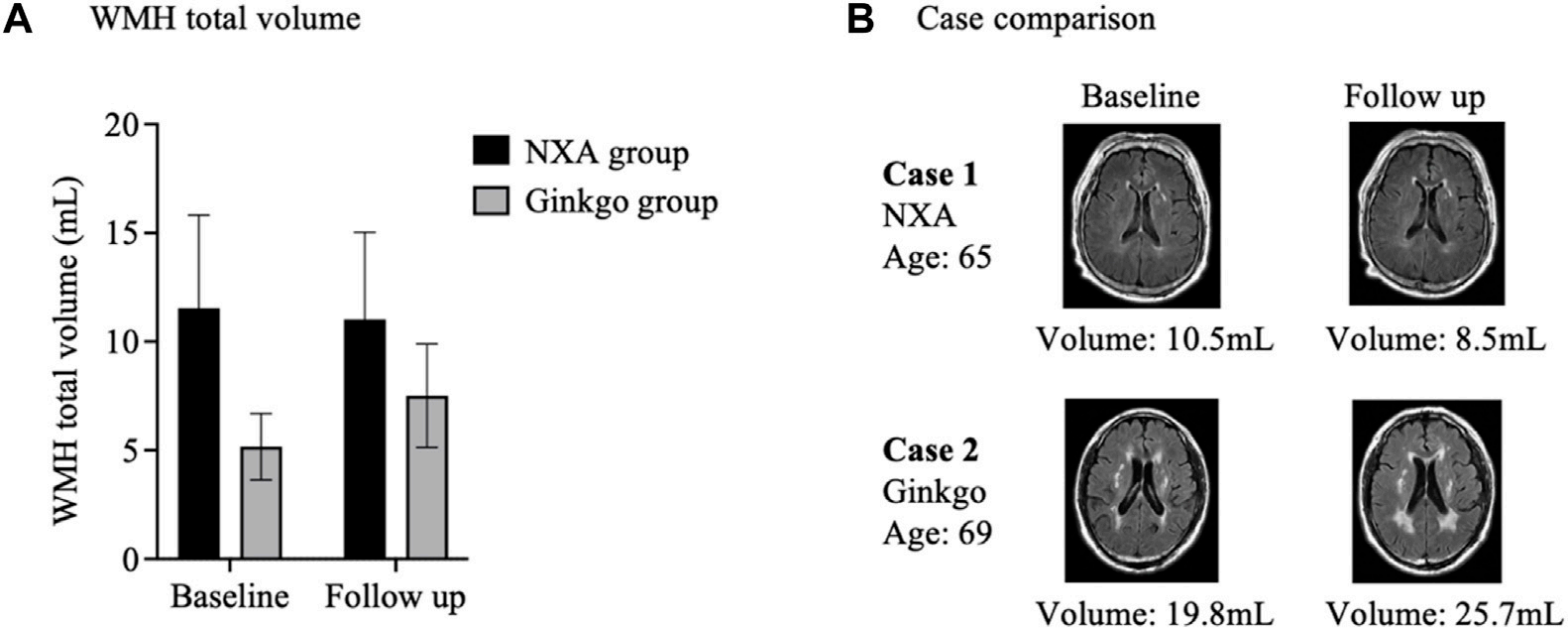
Effects of Naoxin’an capsule on white matter hyperintensity in patients with vascular cognitive impairment. **(A)** After treatment, the total volume of white matter hyperintensity showed a slight decrease in the NXA group and an increase in the ginkgo group but was not statistically significant (*p* > 0.05). **(B)** White matter hyperintensity lesions in individuals in the NXA group and ginkgo group before and after treatment. A 65-year-old male (baseline MMSE: 26) had a decrease in the total volume of white matter hyperintensity from 10.5 mL to 8.5 mL after Naoxin’an capsule treatment for 24 weeks. A 69-year-old male in the ginkgo group (baseline MMSE: 24) had an increase in white matter hyperintensity volume from 19.8 mL to 25.7 mL over the same time.

After treatment, the total volume of WMH showed a slight decrease in the NXA group and an increase in the ginkgo group (Figure 5) but was not statistically significant (*p* > 0.05).

### 3.5 Relationship between altered brain function and cognitive performance

**FIGURE 6:**
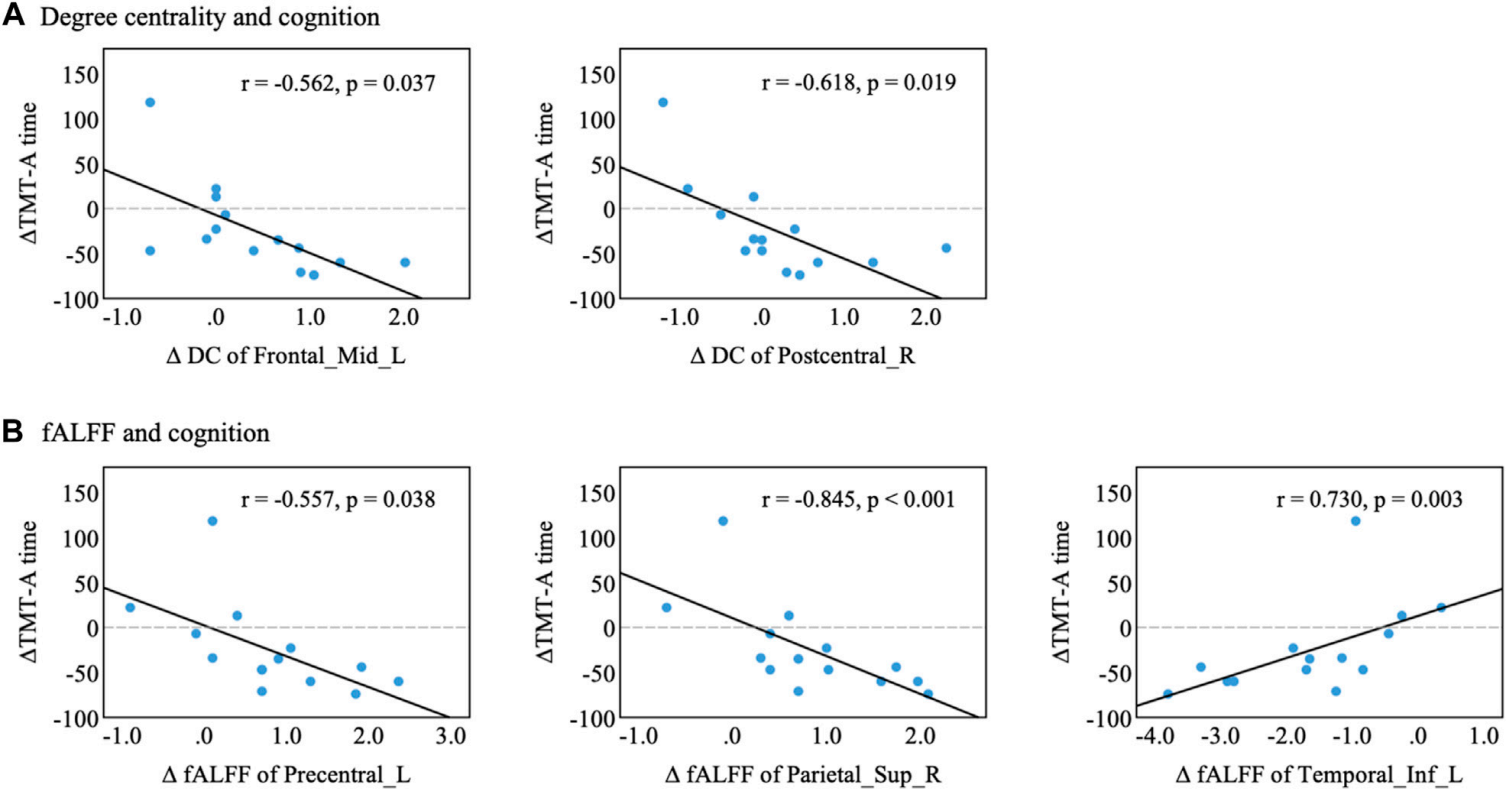
Correlation between altered brain function and cognitive performance in the NXA group. **(A)** Changes in degree centrality were associated with cognitive decline. **(B)** Changes in fALFF were associated with cognitive decline. Changes in index variability were computed as the difference (∆index = index follow‐up‐index baseline) between baseline and follow-up for each participant. Abbreviations: Mid = middle, Sup = superior, Inf = inferior, L = left, R = right.

Finally, we examined the associations of treatment-related changes in brain function (i.e., DC and fALFF with significant interaction) with changes in cognitive function. After adjusting for age, education, and sex, partial correlation results showed that the changes in DC in the left middle frontal gyrus (Figure 6, *p* = 0.037) and right postcentral gyrus (*p* = 0.019) were negatively correlated with the change in TMT-A time in the NXA group. In addition, increased fALFF in the left postcentral gyrus (*p* = 0.038) and right superior parietal gyrus (*p* < 0.001) and decreased fALFF in the left inferior temporal gyrus (*p* = 0.003) after treatment in the NXA group were positively correlated with decreased TMT-A time scores. These findings suggest that the improvement in executive function in the NXA group is associated with changes in brain function.

## 4 Discussion

This study investigated the effectiveness of Naoxin’an capsules on whole-brain spontaneous activity and structural properties in VCI patients. The relationship between alterations in brain regions and cognitive neuropsychological indicators was also evaluated. Results suggested that the NXA capsules can effectively improve the cognitive function of VCI patients, including global cognitive ability, episodic memory, and executive function, after 24 weeks of treatment. The NXA capsules also improved multiregional brain function and increased gray matter density in the left postcentral gyrus. The effect of the NXA capsules on WMH was limited and showed a trend of inhibition. Finally, changes in executive function were closely related to changes in drug-related indices of brain function was detected. These findings suggested that the NXA capsules can improve spontaneous local brain activity, thereby improving the cognitive function of VCI patients. Therefore, the NXA capsules can be used as an effective drug to improve cognitive dysfunction caused by ischemic stroke and have great application potential in the treatment of VCI. This study demonstrated that the efficacy of NXA capsules on VCI through brain regional activity and structural properties analysis was feasible and innovative.

The leading cause of VCI is chronic cerebral hypoperfusion, and cerebral metabolism is closely related to cerebral blood flow (CBF) (Claassen et al., 2021). CBF changes induced by neural activity are required to meet the increased metabolic needs of active brain regions, which require a well-timed delivery of oxygen and glucose (Iadecola, 2017). Glucose is the major energy source for the brain (Dienel, 2019), and the brain needs a continuous energy supply in the form of ATP. The energy generated by oxidative phosphorylated glucose in mitochondria accounts for the majority and is supplemented by aerobic glycolysis in cytoplasm (Cunnane et al., 2020). By activating the CREB/PGC-1α pathway in VCI rats, NXA capsules improve mitochondrial inner membrane integrity, increase mitochondrial membrane potential (MMP), reduce mitochondrial swelling and microscopic damage, and reduce reactive oxygen species (ROS) overreliance induced by chronic cerebral ischemia (Yu-juan, 2022). Mitochondria are a major source of ROS and produce energy in the form of ATP, both of which are closely related to the pathogenesis of cerebral ischemia (Yang et al., 2018). This finding indicated that the NXA capsules could improve cognitive function by regulating the brain energy metabolism in VCI rats.

Solid evidence showed that the hemodynamic features of the voxel-based BOLD signal at rest can reflect the potential metabolic demand, and fALFF can be regarded as a metabolic indicator (Bernier et al., 2017; Deng et al., 2022). Baseline blood flow information in fALFF is primarily attributable to metabolic utilization, for instance, the glucose or oxygen metabolic rate (Deng et al., 2022). The glucose consumption cerebral metabolic rate reflects the energy demand during glucose oxidative phosphorylation, and in part of that matches the metabolic rate of oxygen consumption and glycolysis processes (Me and Ma, 2006). Similar to the current findings, several studies have reported changes in local functional properties in patients with brain ischemia (Guo et al., 2014; Tsai et al., 2014; Shi et al., 2017). Therefore, these local functional properties reflect the pathological manifestations of VCI and can serve as biomarkers to monitor the therapeutic effect of NXA.

The current study further found that NXA capsules increased the fALFF of the right angular and median cingulate gyri, and the right parahippocampal and left inferior temporal gyrus decreased. While cognitive function depends on an advanced integrated system, each brain area plays a distinctive role in a variety of different cognitive domains. As part of a wider lateral parietal cortex system, the function of the angular gyrus is highly related to episodic memory and semantic memory (Humphreys et al., 2021). Also, the mid-cingulate cortex integrates intrinsic brain network signals related to intersensory, allogeneic, executive function, motor planning, and sensory integration (Touroutoglou et al., 2020). Parahippocampal cortex activity dedicates to the encoding and retrieval of episodic memory and visuospatial processing (Aminoff et al., 2013). The temporal cortex is an essential part of the ventral visual pathway and is particularly important for visual processing and visual object recognition (Conway, 2018; Rajalingham et al., 2020). The distribution of aerobic glycolysis in the healthy human brain is different. The glycolysis in the bilateral prefrontal cortex, bilateral lateral parietal lobe, and posterior cingulate gyrus/anterior cuneiform lobe is significantly higher, while the level of aerobic glycolysis in the cerebellum and bilateral inferior temporal gyrus is significantly lower (Vaishnavi et al., 2010). The patterns and regions of fALFF changes after NXA treatment were similar to the aerobic glycolysis in the healthy human brain. Thus, the NXA capsules may modulate cognitive functions by altering the metabolism of these regions.

In addition, current studies showed that NAX capsules increased DC in VCI patients in the middle frontal gyrus, postcentral gyrus, and supplementary motor area, regions that are susceptible to stroke. Node’s DC can measure the communication between brain regions required for information exchange (Zuo et al., 2012). DC can reflect the node characteristics of intrinsic connectivity networks (ICNs). The larger DC in the brain region, the greater its role in information processing. *In vivo* evidence of neuroimaging suggested that VCI may be a network disorder (Iadecola et al., 2019). The frontal lobe is closely related to higher brain functions (executive and memory), and the postcentral gyrus and supplementary motor area, important areas of the brain responsible for proprioception, are at risk of being damaged by stroke (DiGuiseppi and Tadi, 2022). These results suggested that NXA capsules enhance the ability of these regions to transmit information to other brain regions.

Impaired executive function and processing speed are common features in patients with vascular brain lesions (Iadecola et al., 2019). As an indicator of cognitive processing speed and executive function, TMT is widely used in neuropsychological assessment (Sánchez-Cubillo et al., 2009). After 24 weeks of NXA capsules treatment, we found that changes of DC in the left middle frontal gyrus and right retrocentral gyrus, and changes of fALFF in the left retrocentral gyrus, right parietal gyrus, and left inferior temporal gyrus were correlated with changes in TMT-A time. These brain regions, especially the frontal and parietal gyrus, are key to the executive control network (Niendam et al., 2012), which supports a broad range of executive functions. In addition, a previous study demonstrated that spontaneous brain activity in rs-fMRI is associated with the severity of executive dysfunction in patients with VCI (Lei et al., 2014). This evidence supports the current research findings to some extent, the NXA capsules may improve the executive function of VCI patients by altering the metabolism of some brain regions and enhancing the transmission capacity of other brain regions to improve executive function, thereby alleviating the cognitive function of VCI patients.

Cognition in the brain has two complementary principles: functional specialization and dynamic integration. Recent MRI studies have focused on dynamic integration, which relies on the structural topology of brain networks and the dynamics of functional connectivity (de Pasquale et al., 2018). This study investigated the mechanism of action of the NXA capsules through functional magnetic resonance imaging. Results showed that local functional properties, such as ALFF/fALFF, and local structural properties revealed relevant alteration with changed cognitive performance induced by NXA capsules. Thus, local functional properties were also used to assess treatment outcomes. Considering that the structure-function coupling in our brain is disrupted by cognitive impairment (Cocchi et al., 2014; Baum et al., 2020; Cao et al., 2020; Kulik et al., 2022), it is critical for future research to combine the dynamic integration of structural topology, local functional properties, and functional connectomes to further evaluate the therapeutic effect of NXA capsules.

## 5 Conclusion

In conclusion, this study indicated that Naoxin’an capsules improved cognitive impairment, multiregional brain function, and gray matter structure of the postcentral gyrus in VCI patients.

## Data Availability

The original contributions presented in the study are included in the article/Supplementary Material, further inquiries can be directed to the corresponding authors.

## References

[B1] Aminoff E. M. Kveraga K. Bar M. (2013). The role of the parahippocampal cortex in cognition. Trends Cogn. Sci. 17, 379–390. 10.1016/j.tics.2013.06.009 23850264 PMC3786097

[B2] Baum G. L. Cui Z. Roalf D. R. Ciric R. Betzel R. F. Larsen B. (2020). Development of structure–function coupling in human brain networks during youth. Proc. Natl. Acad. Sci. U. S. A. 117, 771–778. 10.1073/pnas.1912034117 31874926 PMC6955327

[B3] Bernier M. Croteau E. Castellano C. A. Cunnane S. C. Whittingstall K. (2017). Spatial distribution of resting-state BOLD regional homogeneity as a predictor of brain glucose uptake: A study in healthy aging. Neuroimage 150, 14–22. 10.1016/j.neuroimage.2017.01.055 28130193

[B4] Biessels G. J. Despa F. (2018). Cognitive decline and dementia in diabetes mellitus: Mechanisms and clinical implications. Nat. Rev. Endocrinol. 14, 591–604. 10.1038/s41574-018-0048-7 30022099 PMC6397437

[B5] Buckner R. L. Sepulcre J. Talukdar T. Krienen F. M. Liu H. Hedden T. (2009). Cortical hubs revealed by intrinsic functional connectivity: Mapping, assessment of stability, and relation to Alzheimer’s disease. J. Neurosci. 29, 1860–1873. 10.1523/JNEUROSCI.5062-08.2009 19211893 PMC2750039

[B6] Cao R. Wang X. Gao Y. Li T. Zhang H. Hussain W. (2020). Abnormal anatomical rich-club organization and structural–functional coupling in mild cognitive impairment and Alzheimer’s disease. Front. Neurol. 11, 1–17. 10.3389/fneur.2020.00053 32117016 PMC7013042

[B7] Chao-Gan Y. Yu-Feng D. Dparsf Z. (2010). Dparsf: A matlab toolbox for "pipeline" data analysis of resting-state fMRI. Front. Syst. Neurosci. 4, 13. 10.3389/fnsys.2010.00013 20577591 PMC2889691

[B8] Claassen J. A. H. R. Thijssen D. H. J. Panerai R. B. Faraci F. M. (2021). Regulation of cerebral blood flow in humans: Physiology and clinical implications of autoregulation. Physiol. Rev. 101, 1487–1559. 10.1152/physrev.00022.2020 33769101 PMC8576366

[B9] Cocchi L. Harding I. H. Lord A. Pantelis C. Yucel M. Zalesky A. (2014). Disruption of structure-function coupling in the schizophrenia connectome. Neuroimage Clin. 4, 779–787. 10.1016/j.nicl.2014.05.004 24936428 PMC4055899

[B10] Collins R. Armitage J. Parish S. Sleight P. Peto R. (2002). MRC/BHF heart protection study of cholesterol lowering with simvastatin in 20,536 high-risk individuals: A randomised placebo-controlled trial. Lancet 360, 7–22. 10.1016/S0140-6736(02)09327-3 12114036

[B11] Conway B. R. (2018). The organization and operation of inferior temporal cortex. Annu. Rev. Vis. Sci. 4, 381–402. 10.1146/annurev-vision-091517-034202 30059648 PMC6404234

[B12] Cunnane S. C. Trushina E. Morland C. Prigione A. Casadesus G. Andrews Z. B. (2020). Brain energy rescue: An emerging therapeutic concept for neurodegenerative disorders of ageing. Nat. Rev. Drug Discov. 19, 609–633. 10.1038/s41573-020-0072-x 32709961 PMC7948516

[B13] de Pasquale F. Corbetta M. Betti V. della Penna S. (2018). Cortical cores in network dynamics. Neuroimage 180, 370–382. 10.1016/j.neuroimage.2017.09.063 28974453

[B14] Deng S. Franklin C. G. O'Boyle M. Zhang W. Heyl B. L. Jerabek P. A. (2022). Hemodynamic and metabolic correspondence of resting-state voxel-based physiological metrics in healthy adults. Neuroimage 250, 118923. 10.1016/j.neuroimage.2022.118923 35066157 PMC9201851

[B15] Dienel G. A. (2019). Brain glucose metabolism: Integration of energetics with function. Physiol. Rev. 99, 949–1045. 10.1152/physrev.00062.2017 30565508

[B16] DiGuiseppi J. Tadi P. (2022). Neuroanatomy, postcentral gyrus. Treasure Island (FL): StatPearls. 31751015

[B17] Farooq M. U. Min J. Goshgarian C. Gorelick P. B. (2017). Pharmacotherapy for vascular cognitive impairment. CNS Drugs 31, 759–776. 10.1007/s40263-017-0459-3 28786085

[B18] Folstein M. F. Folstein S. E. McHugh P. R. (1975). ‘Mini-mental state’. A practical method for grading the cognitive state of patients for the clinician. J. Psychiatr. Res. 12, 189–198. 10.1016/0022-3956(75)90026-6 1202204

[B19] Gordon N. G. (1972). The Trail making test in neuropsychological diagnosis. J. Clin. Psychol. 28, 167–169. 10.1002/1097-4679(197204)28:2<167::aid-jclp2270280212>3.0.co;2-x 5019979

[B20] Guang-Hui H. (2021). Regulation of naoxin’an capsule on glial cell activation and inflammatory response in rats with chronic cerebral hypoperfusion-induced vascular cognitive impairment. Chin. J. Exp. Traditional Med. Formulae 27, 46–55. 10.13422/j.cnki.syfjx.20211806

[B21] Guo J. Chen N. Wu Q. Chen H. Gong Q. (2014). Regional homogeneity abnormalities in patients with transient ischaemic attack: A resting-state fMRI study. Clin. Neurophysiol. 125, 520–525. 10.1016/j.clinph.2013.08.010 24064249

[B22] Humphreys G. F. Lambon Ralph M. A. Simons J. S. (2021). A unifying account of angular gyrus contributions to episodic and semantic cognition. Trends Neurosci. 44, 452–463. 10.1016/j.tins.2021.01.006 33612312

[B23] Iadecola C. Duering M. Hachinski V. Joutel A. Pendlebury S. T. Schneider J. A. (2019). Vascular cognitive impairment and dementia: JACC scientific Expert panel. J. Am. Coll. Cardiol. 73, 3326–3344. 10.1016/j.jacc.2019.04.034 31248555 PMC6719789

[B24] Iadecola C. (2017). The neurovascular unit coming of age: A journey through neurovascular coupling in health and disease. Neuron 96, 17–42. 10.1016/j.neuron.2017.07.030 28957666 PMC5657612

[B25] Kandiah N. Chan Y. F. Chen C. Dasig D. Dominguez J. Han S. H. (2021). Strategies for the use of Ginkgo biloba extract, EGb 761®, in the treatment and management of mild cognitive impairment in Asia: Expert consensus. CNS Neurosci. Ther. 27, 149–162. 10.1111/cns.13536 33352000 PMC7816207

[B26] Kandiah N. Ong P. A. Yuda T. Ng L. L. Mamun K. Merchant R. A. (2019). Treatment of dementia and mild cognitive impairment with or without cerebrovascular disease: Expert consensus on the use of Ginkgo biloba extract, EGb 761. CNS Neurosci. Ther. 25, 288–298. 10.1111/cns.13095 30648358 PMC6488894

[B27] Koh P. O. (2010). Gingko biloba extract (EGb 761) prevents cerebral ischemia-induced p70S6 kinase and S6 phosphorylation. Am. J. Chin. Med. Gard. City N Y) 38, 727–734. 10.1142/S0192415X10008196 20626058

[B28] Kueper J. K. Speechley M. Montero-Odasso M. (2018). The Alzheimer’s disease assessment scale-cognitive subscale (ADAS-Cog): Modifications and responsiveness in pre-dementia populations. A narrative review. J. Alzheimers Dis. 63, 423–444. 10.3233/JAD-170991 29660938 PMC5929311

[B29] Kulik S. D. Nauta I. M. Tewarie P. Koubiyr I. Dellen E. V. (2022). Structure-function coupling as a correlate and potential biomarker of cognitive impairment in multiple sclerosis. Netw. Neurosci. 16, 339–356. 10.1162/netn_a_00226 PMC9208024 35733434

[B30] le Bars P. L. Katz M. M. Berman N. Itil T. M. Freedman A. M. Schatzberg A. F. (1997). A placebo-controlled, double-blind, randomized trial of an extract of Ginkgo biloba for dementia. North American EGb Study Group. JAMA 278, 1327–1332. 10.1001/jama.278.16.1327 9343463

[B31] Lei Y. Li Y. Ni W. Jiang H. Yang Z. Guo Q. (2014). Spontaneous brain activity in adult patients with moyamoya disease: A resting-state fMRI study. Brain Res. 1546, 27–33. 10.1016/j.brainres.2013.12.022 24380677

[B32] Li W. Z. Wu W. Y. Huang H. Wu Y. Y. Yin Y. Y. (2013). Protective effect of bilobalide on learning and memory impairment in rats with vascular dementia. Mol. Med. Rep. 8, 935–941. 10.3892/mmr.2013.1573 23835946

[B33] Me R. Ma M. (2006). Brain work and brain imaging. Annu. Rev. Neurosci. 29, 449–476. 10.1146/annurev.neuro.29.051605.112819 16776593

[B34] Mok E. H. L. Lam L. C. W. Chiu H. F. K. (2004). Category verbal fluency test performance in Chinese elderly with Alzheimer’s disease. Dement. Geriatr. Cogn. Disord. 18, 120–124. 10.1159/000079190 15211065

[B35] Niendam T. A. Laird A. R. Ray K. L. Dean Y. M. Glahn D. C. Carter C. S. (2012). Meta-analytic evidence for a superordinate cognitive control network subserving diverse executive functions. Cogn. Affect Behav. Neurosci. 12, 241–268. 10.3758/s13415-011-0083-5 22282036 PMC3660731

[B36] Rajalingham R. Kar K. Sanghavi S. Dehaene S. DiCarlo J. J. (2020). The inferior temporal cortex is a potential cortical precursor of orthographic processing in untrained monkeys. Nat. Commun. 11, 3886. 10.1038/s41467-020-17714-3 32753603 PMC7403350

[B37] Regier D. A. Narrow W. E. Kuhl E. A. Kupfer D. J. (2009). The conceptual development of DSM-V. Am. J. Psychiatry 166, 645–650. 10.1176/appi.ajp.2009.09020279 19487400

[B38] Rockwood K. Wentzel C. Hachinski V. Hogan D. B. MacKnight C. McDowell I. (2000). Prevalence and outcomes of vascular cognitive impairment. Vascular cognitive impairment investigators of the Canadian study of health and aging. Neurology 54, 447–451. 10.1212/wnl.54.2.447 10668712

[B39] Román G. C. Tatemichi T. K. Erkinjuntti T. Cummings J. L. Masdeu J. C. Garcia J. H. (1993). Vascular dementia: Diagnostic criteria for research studies. Report of the NINDS-AIREN international workshop. Neurology 43, 250–260. 10.1212/wnl.43.2.250 8094895

[B40] Saleem S. Zhuang H. Biswal S. Christen Y. Doré S. (2008). Ginkgo biloba extract neuroprotective action is dependent on heme oxygenase 1 in ischemic reperfusion brain injury. Stroke 39, 3389–3396. 10.1161/STROKEAHA.108.523480 18845796 PMC6010172

[B41] Sánchez-Cubillo I. Perianez J. A. Adrover-Roig D. Rodriguez-Sanchez J. M. Rios-Lago M. Tirapu J. (2009). Construct validity of the Trail making test: Role of task-switching, working memory, inhibition/interference control, and visuomotor abilities. J. Int. Neuropsychol. Soc. 15, 438–450. 10.1017/S1355617709090626 19402930

[B42] Schmidt P. Gaser C. Arsic M. Buck D. Forschler A. Berthele A. (2012). An automated tool for detection of FLAIR-hyperintense white-matter lesions in Multiple Sclerosis. Neuroimage 59, 3774–3783. 10.1016/j.neuroimage.2011.11.032 22119648

[B43] Shepherd J. Blauw G. J. Murphy M. B. Bollen E. L. E. M. Buckley B. M. Cobbe S. M. (2002). Pravastatin in elderly individuals at risk of vascular disease (prosper): A randomised controlled trial. Lancet 360, 1623–1630. 10.1016/s0140-6736(02)11600-x 12457784

[B44] Shi Y. Zeng Y. Wu L. Liu Z. Zhang S. Yang J. (2017). A study of the brain functional network of post-stroke depression in three different lesion locations. Sci. Rep. 7, 14795. 10.1038/s41598-017-14675-4 29093543 PMC5665859

[B45] Strømgaard K. Nakanishi K. (2004). Chemistry and biology of terpene trilactones from Ginkgo biloba. Angew. Chem. Int. Ed. Engl. 43, 1640–1658. 10.1002/anie.200300601 15038029

[B46] Tchantchou F. Xu Y. Wu Y. Christen Y. Luo Y. (2007). EGb 761 enhances adult hippocampal neurogenesis and phosphorylation of CREB in transgenic mouse model of Alzheimer’s disease. FASEB J. 21, 2400–2408. 10.1096/fj.06-7649com 17356006

[B47] Touroutoglou A. Andreano J. Dickerson B. C. Barrett L. F. (2020). The tenacious brain: How the anterior mid-cingulate contributes to achieving goals. Cortex 123, 12–29. 10.1016/j.cortex.2019.09.011 31733343 PMC7381101

[B48] Tsai Y. H. Yuan R. Huang Y. C. Yeh M. Y. Lin C. P. Biswal B. B. (2014). Disruption of brain connectivity in acute stroke patients with early impairment in consciousness. Front. Psychol. 4, 956. 10.3389/fpsyg.2013.00956 24427147 PMC3877750

[B49] Tupler L. A. Welsh K. A. Asare-Aboagye Y. Dawson D. V. (1995). Reliability of the rey-osterrieth Complex figure in use with memory-impaired patients. J. Clin. Exp. Neuropsychol. 17, 566–579. 10.1080/01688639508405146 7593476

[B50] Vaishnavi S. N. Vlassenko A. G. Rundle M. M. Snyder A. Z. Mintun M. A. Raichle M. E. (2010). Regional aerobic glycolysis in the human brain. Proc. Natl. Acad. Sci. U. S. A. 107, 17757–17762. 10.1073/pnas.1010459107 20837536 PMC2955101

[B51] van der Flier W. M. Skoog I. Schneider J. A. Pantoni L. Mok V. Chen C. L. H. (2018). Vascular cognitive impairment. Nat. Rev. Dis. Prim. 4, 18003. 10.1038/nrdp.2018.3 29446769

[B52] Wang H. Yu X. Li S. Chen Y. Li H. He J. (2004). The cognitive subscale of Alzheimer’s Disease Assessment Scale, Chinese version in staging of Alzheimer disease. Alzheimer Dis. Assoc. Disord. 18, 231–235. 10.2217/bmm.12.58 15592136

[B53] Xi Q. Zhao X. Wang P. Guo Q. Jiang H. Cao X. (2012). Spontaneous brain activity in mild cognitive impairment revealed by amplitude of low-frequency fluctuation analysis: A resting-state fMRI study. Radiol. Med. 117, 865–871. 10.1007/s11547-011-0780-8 22241376

[B54] Yang J. L. Mukda S. Chen S. D. (2018). Diverse roles of mitochondria in ischemic stroke. Redox Biol. 16, 263–275. 10.1016/j.redox.2018.03.002 29549824 PMC5854930

[B55] Yoshitake T. Yoshitake S. Kehr J. (2010). The Ginkgo biloba extract EGb 761(R) and its main constituent flavonoids and ginkgolides increase extracellular dopamine levels in the rat prefrontal cortex. Br. J. Pharmacol. 159, 659–668. 10.1111/j.1476-5381.2009.00580.x 20105177 PMC2828029

[B56] Yu-juan F. (2022). Naoxin’an capsule improves mitochondrial and oxidative damage in chronic cerebral ischemia induced VCI rats via activating CREB/PGC-1α pathway. Chin. J. Exp. Traditional Med. Formulae 28 (23), 19–29. 10.13422/j.cnki.syfjx.20221306

[B57] Zang Y. Jiang T. Lu Y. He Y. Tian L. (2004). Regional homogeneity approach to fMRI data analysis. Neuroimage 22, 394–400. 10.1016/j.neuroimage.2003.12.030 15110032

[B58] Zhang X. Su J. Gao C. Ni W. Gao X. Li Y. (2019). Progression in vascular cognitive impairment: Pathogenesis, neuroimaging evaluation, and treatment. Cell. Transpl. 28, 18–25. 10.1177/0963689718815820 PMC6322135 30488737

[B59] Zhu J. D. Wang J. J. Zhang X. H. Yu Y. Kang Z. S. (2018). Panax ginseng extract attenuates neuronal injury and cognitive deficits in rats with vascular dementia induced by chronic cerebral hypoperfusion. Neural Regen. Res. 13, 664–672. 10.4103/1673-5374.230292 29722318 PMC5950676

[B60] Zonneveld T. P. Richard E. Vergouwen M. D. Nederkoorn P. J. de Haan R. Roos Y. B. (2018). Blood pressure-lowering treatment for preventing recurrent stroke, major vascular events, and dementia in patients with a history of stroke or transient ischaemic attack. Cochrane Database Syst. Rev. 7, CD007858. 10.1002/14651858.CD007858.pub2 30024023 PMC6513249

[B61] Zuo X. N. Ehmke R. Mennes M. Imperati D. Castellanos F. X. Sporns O. (2012). Network centrality in the human functional connectome. Cereb. Cortex 22, 1862–1875. 10.1093/cercor/bhr269 21968567

