# Supplementary material for: Naoxin’an capsules protect brain function and structure in patients with vascular cognitive impairment
Source: Front Pharmacol. 2023 Apr 5;14:1129125. doi: 10.3389/fphar.2023.1129125 (PMC10113453; doi:10.3389/fphar.2023.1129125)
Supplement: Supplementary file 1 [file DataSheet1.docx]

**Supplementary material**

**Diagnostic Criteria of VCI**

The diagnostic criteria of VCI refer to the Diagnostic and Statistical Manual of Mental Disorders, Fifth Edition (DSM-V)^[1]^ or National Institute of Neurological Disorders and Stroke-Association Internationale pour la Recherche et l’Enseignement en Neurosciences (NINDS-AIREN) criteria^[2]^: (1) Hachinski Ischemia Scale (HIS) score > 4; (2) Evidence of cerebrovascular disease from history, physical examination, and/or neuroimaging that is considered sufficient to account for the neurocognitive deficits; (3) Evidence of modest cognitive decline from a previous level of performance in at least one cognitive domains (complex attention, executive function, learning and memory, language, perceptual-motor or social cognition) based on.

**Supplementary Table 1.** **Effect of NXA capsule on cognitive performance in younger patients with vascular cognitive impairment.**

|  | | **NXA group** | | **Ginkgo group** | | | **Interactions** | | |
| --- | --- | --- | --- | --- | --- | --- | --- | --- | --- |
|  | **Baseline** | | **Follow up** | | **Baseline** | **Follow up** | | **F** | **p** |
| ***Main outcomes*** | | | | | | | | | |
| ADAS-Cog | 9.18±3.795 | | 6.29±2.654 | | 7.59±1.503 | 6.63±1.716 | | 8.805 | **0.005** |
| MMSE | 27.26±2.435 | | 28.03±2.111 | | 26.88±1.453 | 26.81±2.060 | | 3.265 | 0.079 |
| ***Episodic memory*** | | | | | | | | | |
| AVLT N1-N5 | 25.83±9.523 | | 28.52±9.853 | | 30.71±6.430 | 29.88±5.384 | | 2.853 | 0.100 |
| RO-CFT Recall | 9.91±6.674 | | 11.04±6.456 | | 15.18±6.975 | 13.76±5.333 | | 7.208 | **0.011** |
| ***Visual-spatial*** | | | | | | | | | |
| RO-CFT Copy | 27.65±6.072 | | 25.95±8.250 | | 31.18±5.812 | 31.24±5.548 | | 2.06 | 0.160 |
| ***Executive function*** | | | | | | | | | |
| TMT-A | 110.0±120.059 | | 66.71±61.065 | | 56.53±18.170 | 54.34±17.833 | | 13.965 | **0.001** |
| ***Language function*** | | | | | | | | | |
| CVFT | 39.96±8.921 | | 38.67±12.007 | | 44.41±8.544 | 44.43±8.410 | | 0.353 | 0.556 |

Abbreviation: ADAS-cog = Alzheimer’s Disease Assessment Scale-Cognitive subscale; MMSE = Mini-Mental Status Examination; AVLT = Auditory Verbal Learning Test; RO-CFT = Rey-Osterrieth Complex Figure test; CVFT = Category Verbal Fluency Test; TMT = Trail Making Test.

**Supplementary Table 2. Effect of NXA capsule on cognitive performance in older patients with vascular cognitive impairment.**

|  | | **NXA group** | | **Ginkgo group** | | | **Interactions** | | |
| --- | --- | --- | --- | --- | --- | --- | --- | --- | --- |
|  | **Baseline** | | **Follow up** | | **Baseline** | **Follow up** | | **F** | **p** |
| ***Main outcomes*** | | | | | | | | | |
| ADAS-Cog | 9.67±3.355 | | 7.60±2.551 | | 9.82±3.445 | 10.03±3.687 | | 7.346 | **0.010** |
| MMSE | 27.18±2.922 | | 27.46±2.434 | | 26.00±1.940 | 25.23±1.512 | | 3.447 | 0.072 |
| ***Episodic memory*** | | | | | | | | | |
| AVLT N1-N5 | 23.82±7.563 | | 27.14±6.985 | | 24.00±7.600 | 24.17±6.392 | | 8.235 | **0.007** |
| RO-CFT Recall | 7.18±4.905 | | 10.18±4.876 | | 9.11±5.508 | 8.72±4.956 | | 13.143 | **0.001** |
| ***Visual-spatial*** | | | | | | | | | |
| RO-CFT Copy | 23.05±6.440 | | 24.02±7.418 | | 29.94±5.385 | 29.44±4.932 | | 3.272 | 0.079 |
| ***Executive function*** | | | | | | | | | |
| TMT-A | 119.86±122.804 | | 92.89±76.698 | | 70.61±37.007 | 70.17±37.690 | | 1.281 | 0.265 |
| ***Language function*** | | | | | | | | | |
| CVFT | 38.95±11.013 | | 38.16±11.157 | | 40.56±8.234 | 40.51±8.553 | | 1.026 | 0.318 |

Abbreviation: ADAS-cog = Alzheimer’s Disease Assessment Scale-Cognitive subscale; MMSE = Mini-Mental Status Examination; AVLT = Auditory Verbal Learning Test; RO-CFT = Rey-Osterrieth Complex Figure test; CVFT = Category Verbal Fluency Test; TMT = Trail Making Test.

**Supplementary Table 3.** **Effect of NXA capsule on cognitive performance in male patients with vascular cognitive impairment.**

|  | **NXA group** | | **Ginkgo group** | | **Interactions** | |
| --- | --- | --- | --- | --- | --- | --- |
|  | **Baseline** | **Follow up** | **Baseline** | **Follow up** | **F** | **p** |
| ***Main outcomes*** | | | | | | |
| ADAS-Cog | 10.92±3.976 | 7.78±2.929 | 8.94±3.258 | 8.39±3.898 | 10.76 | **0.002** |
| MMSE | 26.24±3.045 | 27.08±2.525 | 26.56±1.886 | 26.14±1.972 | 9.158 | **0.004** |
| ***Episodic memory*** | | | | | | |
| AVLT N1-N5 | 21.44±8.312 | 24.68±8.877 | 25.39±7.293 | 25.33±6.117 | 3.526 | 0.068 |
| RO-CFT Recall | 7.00±5.346 | 8.60±5.025 | 12.06±7.557 | 11.11±6.398 | 4.968 | **0.032** |
| ***Visual-spatial*** | | | | | | |
| RO-CFT Copy | 24.52±7.024 | 23.71±8.738 | 28.72±6.807 | 28.79±6.432 | 0.757 | 0.39 |
| ***Executive function*** | | | | | | |
| TMT-A | 139.40±151.783 | 82.65±74.727 | 61.83±23.355 | 58.02±23.673 | 3.671 | 0.063 |
| ***Language function*** | | | | | | |
| CVFT | 34.84±10.788 | 33.36±13.049 | 42.83±9.642 | 42.84±9.509 | 0.602 | 0.442 |

Abbreviation: ADAS-cog = Alzheimer’s Disease Assessment Scale-Cognitive subscale; MMSE = Mini-Mental Status Examination; AVLT = Auditory Verbal Learning Test; RO-CFT = Rey-Osterrieth Complex Figure test; CVFT = Category Verbal Fluency Test; TMT = Trail Making Test.

**Supplementary Table 4. Effect of NXA capsule on cognitive performance in female patients with vascular cognitive impairment.**

|  | **NXA group** | | **Ginkgo group** | | **Interactions** | |
| --- | --- | --- | --- | --- | --- | --- |
|  | **Baseline** | **Follow up** | **Baseline** | **Follow up** | **F** | **p** |
| ***Main outcomes*** | | | | | | |
| ADAS-Cog | 7.54±1.613 | 5.87±1.845 | 8.52±2.489 | 8.36±2.753 | 6.364 | **0.017** |
| MMSE | 28.45±1.317 | 28.59±1.589 | 26.29±1.649 | 25.84±1.964 | 0.813 | 0.374 |
| ***Episodic memory*** | | | | | | |
| AVLT N1-N5 | 29.10±7.003 | 31.80±6.187 | 29.24±7.918 | 28.65±6.670 | 5.79 | **0.022** |
| RO-CFT Recall | 10.55±6.253 | 13.15±5.566 | 12.06±6.349 | 11.24±4.994 | 14.778 | **0.001** |
| ***Visual-spatial*** | | | | | | |
| RO-CFT Copy | 26.50±6.039 | 26.63±6.355 | 32.47±2.918 | 31.92±3.016 | 0.861 | 0.360 |
| ***Executive function*** | | | | | | |
| TMT-A | 84.10±50.268 | 76.68±65.609 | 65.82±36.130 | 67.21±36.370 | 2.006 | 0.166 |
| ***Language function*** | | | | | | |
| CVFT | 45.25±4.051 | 44.74±3.717 | 42.00±7.348 | 41.96±7.771 | 0.372 | 0.546 |

Abbreviation: ADAS-cog = Alzheimer’s Disease Assessment Scale-Cognitive subscale; MMSE = Mini-Mental Status Examination; AVLT = Auditory Verbal Learning Test; RO-CFT = Rey-Osterrieth Complex Figure test; CVFT = Category Verbal Fluency Test; TMT = Trail Making Test.

**Supplementary table 5. Interaction results for degree centrality and fALFF (GRF-corrected).**

|  | **Number of voxels** | **Peak intensity** | **Peak MNI coordinate** | | |
| --- | --- | --- | --- | --- | --- |
|  |  |  | **x** | **y** | **z** |
| **Degree centrality** | | | | | |
| Frontal_Mid_L | 127 | 20.5775 | -39 | 42 | 15 |
| Postcentral_R | 48 | 28.6981 | 66 | -15 | 21 |
| Supp_Motor_Area_L | 35 | 13.3741 | -12 | -9 | 72 |
| **fALFF** | | | | | |
| ParaHippocampal_R | 2126 | 34.6903 | 27 | 6 | -27 |
| Temporal_Inf_L | 601 | 32.4535 | -45 | -3 | -33 |
| Parietal_Sup_R | 1048 | 30.0461 | 18 | -60 | 63 |
| Precentral_L | 140 | 21.8741 | -30 | -3 | 45 |

Abbreviations: Mid = middle, Inf = inferior, Sup = superior, L = left, R = right.

**Supplementary table 6.** **Paired t-test results for** **degree centrality for pre-and post-treatment (GRF-corrected).**

|  | **Number of voxels** | **Peak**  **intensity** | **Peak MNI coordinate** | | |
| --- | --- | --- | --- | --- | --- |
|  |  |  | **x** | **y** | **z** |
| **NXA group** | | | | | |
| Thalamus_L | 226 | 5.6188 | -15 | -33 | 6 |
| **Ginkgo group** | | | | | |
| Cerebelum_9_L | 271 | 4.5478 | -18 | -42 | -45 |
| Frontal_Inf_Tri_L | 259 | -5.0505 | -39 | 45 | 12 |

Abbreviations: Inf = inferior, Tri = Triangular, L = left, R = right.

**Supplementary table 7. Paired t-test results for fALFF for pre-and post-treatment (GRF-corrected).**

|  | **Number of voxels** | **Peak intensity** | **Peak MNI coordinate** | | |
| --- | --- | --- | --- | --- | --- |
|  |  |  | **x** | **y** | **z** |
| **NXA group** | | | | | |
| Cerebelum_4_5_R | 1232 | -6.5339 | 21 | -33 | -30 |
| Temporal_Pole_Sup_R | 362 | -6.75 | 54 | 12 | -12 |
| Frontal_Med_Orb_R | 444 | -6.0654 | 9 | 45 | -9 |
| Angular_R | 211 | 6.4397 | 45 | -75 | 42 |
| Cingulum_Mid_R | 136 | 5.8485 | 15 | -21 | 45 |
| **Ginkgo group** | | | | | |
| Cerebelum_10_L | 128 | 5.5397 | -12 | -36 | -48 |
| Temporal_Pole_Mid_R | 184 | 5.3406 | 45 | 12 | -36 |
| Rolandic_Oper_L | 316 | 5.3256 | -54 | -3 | 12 |
| Cerebelum_4_5_R | 154 | 5.2156 | 6 | -42 | -33 |
| Temporal_Inf_L | 144 | 4.7937 | -51 | -54 | -15 |
| Parietal_Inf_R | 979 | -5.1406 | 54 | -48 | 54 |

Abbreviations: Sup = superior, Orb = orbital, Med = medial, Inf = inferior, Mid = middle, Oper = operculum, L = left, R = right.

**Supplementary table 8. Paired t-test results for GMV for pre-and post-treatment (GRF-corrected).**

|  | **Number of voxels** | **Peak**  **intensity** | **Peak MNI coordinate** | | |
| --- | --- | --- | --- | --- | --- |
|  |  |  | **x** | **y** | **z** |
| **NXA group** | | | | | |
| Postcentral_L | 215 | 5.1417 | -52.5 | -7.5 | 16.5 |

Abbreviations: L = left.


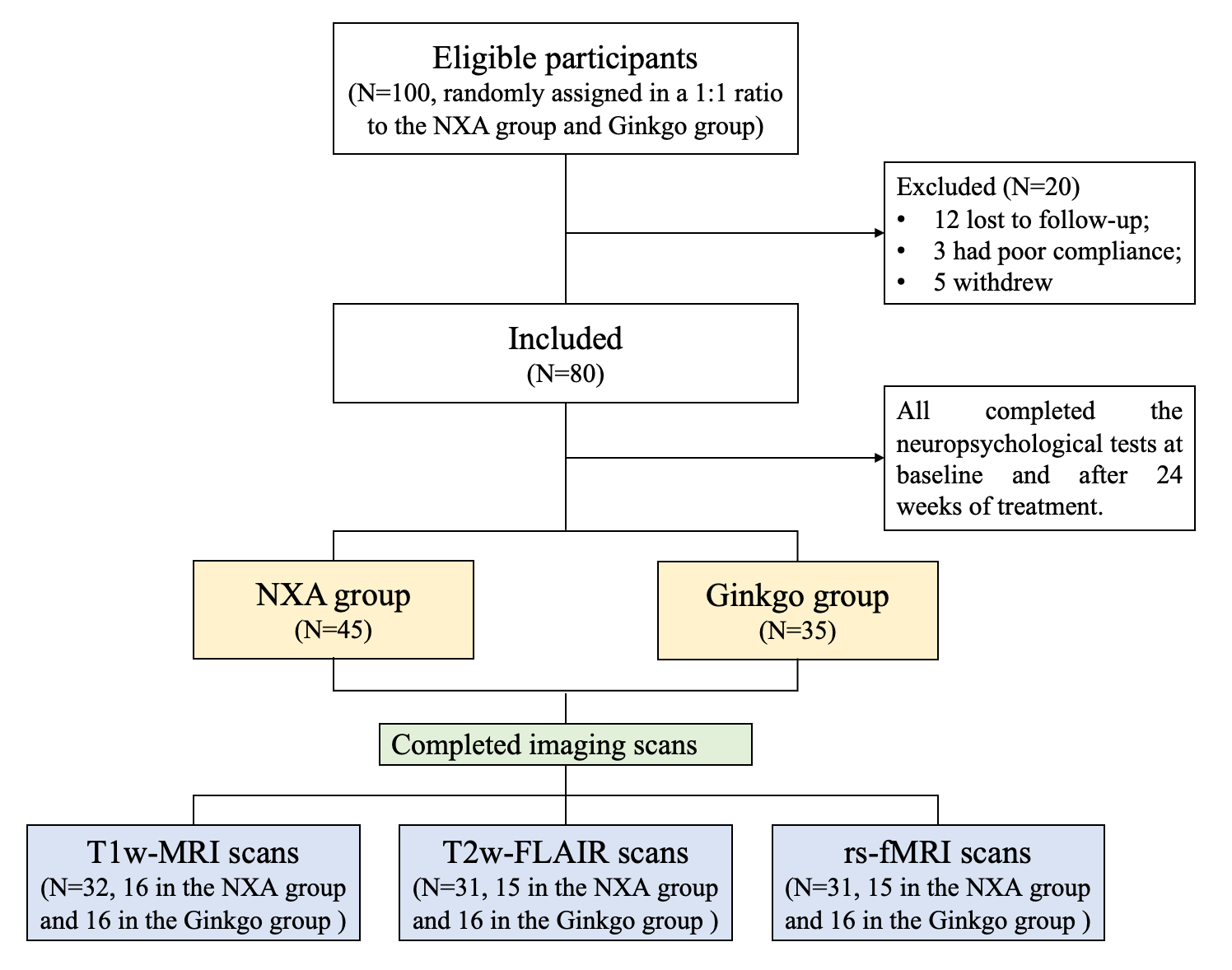


**Supplementary Figure 1. The inclusion process of participants.**

**References**

1 Regier DA, Narrow WE, Kuhl EA, Kupfer DJ. The conceptual development of DSM-V. American Journal of Psychiatry. 2009;166(6):645-50.

2 Román GC, Tatemichi TK, Erkinjuntti T, Cummings J, Masdeu J, Garcia J, et al. Vascular dementia: diagnostic criteria for research studies: report of the NINDS‐AIREN International Workshop. Neurology. 1993;43(2):250-50.
